# Anemia and Transfusion in Infective Endocarditis

**DOI:** 10.31083/RCM33394

**Published:** 2025-06-23

**Authors:** Tim Alberts, Susanne Eberl, Thomas W. van der Vaart, S. Matthijs Boekholdt, Henning Hermanns

**Affiliations:** ^1^Department of Anesthesiology, Amsterdam UMC, University of Amsterdam, 1105 AZ Amsterdam, The Netherlands; ^2^Department of Internal Medicine, Division of Infectious Diseases, Amsterdam UMC, University of Amsterdam, 1105 AZ Amsterdam, The Netherlands; ^3^Department of Cardiology, Amsterdam Cardiovascular Sciences, Amsterdam UMC, University of Amsterdam, 1105 AZ Amsterdam, The Netherlands

**Keywords:** infective endocarditis, anemia, blood transfusion, cardiac surgery, anesthesia

## Abstract

Infective endocarditis (IE) is a severe condition characterized by a predominantly bacterial infection of the heart valves or endocardial surface, often leading to significant morbidity and mortality. Anemia is very common in patients with IE, which may be explained by factors such as chronic inflammation, hemolysis, kidney disease, and pre-existing iron deficiency. This review aimed to comprehensively examine the prevalence, causes, and clinical impact of anemia in IE patients and the role of blood transfusion in managing these patients. The diagnostic approach to anemia in IE includes combining clinical assessment and laboratory investigations, specifically distinguishing between different etiologies. Blood transfusion is likewise very common in IE, especially in surgically treated patients. Thus, balancing the need to correct anemia with the risks associated with blood transfusion is complex, and robust evidence is scarce. Management strategies for anemia in IE may extend beyond transfusion, encompassing pharmacological treatments such as iron supplementation and erythropoiesis-stimulating agents. Despite advancements in understanding the interplay between anemia and IE, several knowledge gaps and unresolved questions remain, necessitating further research to refine treatment protocols and improve patient outcomes. Future directions include investigating emerging therapeutic approaches, optimizing multidisciplinary care pathways, and developing evidence-based guidelines tailored to the unique needs of IE patients. This review underscores the importance of a comprehensive, individualized approach to managing anemia and transfusion in IE, aiming to enhance clinical outcomes and quality of life for affected patients.

## 1. Introduction

Infective endocarditis (IE) is an infrequent but life-threatening disease characterized by an infection of the endocardial surface of the heart, typically affecting the native or prosthetic heart valves [1]. Despite advancements in medical and surgical treatment, IE remains associated with high morbidity and mortality, necessitating multidisciplinary management [2]. The fact that anemia is frequently observed in patients with IE, has been acknowledged for decades [3], but progress in the evidence on this topic is limited, even in the recent literature. The interplay between anemia and IE is complex and multifaceted, likely impacting patient outcomes and complicating management strategies, particularly in the perioperative setting [4, 5]. The potential impact of anemia on adverse outcome has only been addressed very recently [6].

The management of anemia in IE involves a combination of diagnostic evaluation, therapeutic interventions, and often, blood transfusion. Transfusion practices in this context are particularly challenging due to the need to balance the correction of anemia with the potential risks associated with blood transfusion, such as transfusion reactions, immunomodulation, and increased susceptibility to infections [7]. Furthermore, the optimal transfusion thresholds and strategies for this patient population remain unclear.

The primary objective of this narrative review is to provide a comprehensive overview of the current understanding of anemia and transfusion in the context of IE. This includes an examination of the pathophysiology, incidence, and clinical impact of anemia in IE patients, as well as a critical evaluation of transfusion practices and their implications for patient outcomes.

## 2. Literature Review

### 2.1 Mechanisms of Anemia in Infective Endocarditis

While the exact mechanisms remain to be elucidated, anemia in IE most likely results from various, often interrelated mechanisms. Important factors include inflammation and hemolysis, often compounded by the presence of comorbid conditions such as chronic kidney disease and iron deficiency, which can further exacerbate the severity of anemia. As follows, we detail on some of the presumably relevant factors contributing to anemia in IE.

#### 2.1.1 Inflammation

In systemic inflammation, several immune mechanisms account for inflammatory anemia, whereby both red blood cell (RBC) production and clearance are affected [8]. IE triggers a systemic inflammatory response, marked by elevated levels of inflammatory cytokines such as interleukin (IL)-6 [9]. Consecutively, those circulating inflammatory cytokines effectuate anemia via inflammation-driven iron restriction, erythropoietic suppression, and reduced erythrocyte survival.

Iron restriction, mediated by the overproduction of hepcidin, is driven by cytokines such as IL-6. Hepcidin blocks iron export by degrading ferroportin, trapping iron in macrophages, and reducing dietary iron absorption. This leads to a decrease in iron availability for erythropoiesis. Moreover, inflammatory cytokines like tumor necrosis factor (TNF) and IL-1 directly suppress erythropoietic activity, further contributing to anemia. These cytokines decrease erythropoietin (Epo) production and its signaling, limiting RBC production despite the body’s need to compensate for low hemoglobin levels. Inflammatory damage to erythroid progenitors exacerbates this suppression, resulting in blunted responses to Epo [10].

The lifespan of erythrocytes is also shortened due to increased erythrophagocytosis, where activated macrophages destroy RBCs more rapidly. In acute inflammation, such as during severe infection or sepsis, this accelerated RBC destruction leads to anemia within days, beyond what can be attributed to iron sequestration alone [11]. Furthermore, in an experimental setting, splenic enlargement, accompanied by RBC sequestration was shown to contribute to anemia in IE [12].

#### 2.1.2 Hemolysis

Hemolysis, or the destruction of RBCs, is another contributing factor to anemia in IE. Hemolysis can be caused by several mechanisms, including the mechanical damage to RBCs by preexisting valve stenosis, vegetations on heart valves, the presence of prosthetic heart valves, paravalvular leakage of prosthetic heart valves or the immune-mediated destruction of RBCs, as described above [13, 14]. Signs of hemolysis include increased concentration of fragmented erythrocytes (schistocytes) due to mechanical destruction, whereas an immune-mediated cause is suggested by the detection of spherocytes and a positive direct Coombs test. Management options may include surgery to exclude vegetations and paravalvular leakage. Furthermore, there is published literature on the use of beta blocker therapy leading to reduction of the pressure gradient in a case of IE with left ventricular outflow tract stenosis and an improvement of the hemolytic anemia [15].

#### 2.1.3 Comorbid Conditions

Iron deficiency is common in the general population [16], and the prevalence is even higher in patients with cardiovascular disease such as heart failure and aortic valve disease (up to 50%) [17]. The precise percentage of patients with IE that have iron deficiency anemia is not known, though it is conceivable that iron deficiency plays a role in IE-related anemia in a subset of patients, while the exact contribution remains to be elucidated.

Chronic kidney disease, prevalent in about a third of IE patients [18], results in decreased Epo production, impairing the stimulation of RBC production. Furthermore, patients undergoing dialysis experience additional blood loss and hemolysis, exacerbating anemia.

More than 10% of patients with IE have cancer [19]. In those patients, cancer-related anemia can result from bone marrow infiltration, chemotherapy, or chronic disease-related inflammation [20].

#### 2.1.4 Iatrogenic Mechanisms

Surgical interventions to treat complications such as embolectomies and, in particular, cardiac surgery for valve replacement or repair, increase the risk of bleeding, leading to acute blood loss and worsening anemia. IE affects the coagulation system in general [21] and patients with IE undergoing cardiac surgery have more bleeding complications compared to non-IE patients [22]. This blood loss can potentially be exacerbated by the use of anticoagulants and antiplatelet agents. Further sources for iatrogenic blood loss include frequent blood drawings, especially in the critically ill, or the initiation of renal replacement therapy due to acute kidney injury [23].

#### 2.1.5 Other Mechanisms

Other contributing factors include malnutrition and the presence of mechanical heart valves. Although rare, several antibiotics can cause hematological side-effects such as bone marrow suppression or drug-induced immune hemolytic anemia. This includes many classes of the commonly used antibiotics in the treatment of IE, such as cephalosporins, penicillins, carbapenems, rifamycins and oxazolidinones [5]. Such a drug-induced anemia may necessitate a switch in antibiotic therapy.

### 2.2 Epidemiology of Anemia in Infective Endocarditis

Although early studies dating back up to 100 years ago already showed a prevalence of anemia in 74–100% of patients with IE [3, 24], the data on anemia is lacking in many publications in the years thereafter. For instance, several of the largest and most cited cohort studies on IE patients published within the last 15 years, do not report data on anemia [18, 25, 26, 27, 28].

When looking at the studies that did report the prevalence of anemia, there is a large variability, which is largely explained by differences in the definition of anemia, the method of data collection, the population studied and the time of measurement (see Table 1, Ref. [6, 29, 30, 31, 32, 33, 34, 35, 36, 37, 38, 39, 40]). While some investigations defined anemia as a hemoglobin concentration below 90 or 92 g/L, respectively reporting incidences of 16.7–22% [29, 30, 31], this would be considered at least moderate anemia already when applying the World Health Organization (WHO) guideline on hemoglobin cutoffs to define anemia [41].

**Table 1.** **Reported incidence of anemia in IE**.

Author, year	Population, country	Study design	Hemoglobin threshold	Incidence of anemia	Comment
Ferrera *et al*., 2016 [30]	Mixed population, n = 507; Spain	Retrospective multicenter cohort study	90 g/L	21.9%	Patients with left-sided IE, incidence was not different between patients with sinus rhythm or atrial fibrillation
Luo *et al*., 2022 [31]	Mixed population, n = 267; China	Retrospective multicenter cohort study	90 g/L	16.7%	Moderate or severe anemia referred to hemoglobin <90 g/L
Yoshioka *et al*., 2015 [29]	Surgical population, n = 267; Japan	Retrospective multicenter cohort study	92 g/L	32.3%	Preoperative anemia as risk factor for intraoperative intracranial hemorrhagic complications
Alkhouli *et al*., 2019 [33]	Surgical population, n = 34,655; USA	Retrospective database registry	ICD code	32.9%	Patients undergoing mitral valve surgery with IE
Rudasill *et al*., 2019 [32]	Mixed population, n = 123,776; USA	Retrospective database registry	ICD code	35%	Incidence of anemia is higher in iv drug users (38%), and among these, higher in those treated surgically (46.2%)
Agrawal *et al*., 2020 [39]	Mixed population, n = 187,438; USA	Retrospective database registry	ICD code	41%	Anemia as predictor of 30-day re-admission
Mentias *et al*., 2020 [40]	Mixed population, n = 1868; USA	Retrospective database registry	ICD code	50.4%	Patients with IE after transcatheter aortic valve replacement (TAVR), mean age 80.1 ± 9.1 years
Jamal *et al*., 2021 [34]	Mixed population, n = 18,733; USA	Retrospective database registry	ICD code	18%	No difference in anemia incidence between patients with or without heart block
Siddiqui *et al*., 2022 [35]	Mixed population, n = 9029; USA	Retrospective database registry	ICD code	43.5%	Patients with tricuspid valve IE, incidence higher in medically (43.6%), compared to surgically treated patients (38.4%)
Hogan *et al*., 2023 [36]	Surgical population, n = 4206; USA	Retrospective database registry	ICD code	10%	Patients with IE who underwent isolated mitral valve replacement
Lu *et al*., 2013 [37]	Mixed population, n = 148; Australia	Retrospective single center cohort study	WHO criteria	64%	Anemia associated with all-cause long-term mortality
Gatti *et al*., 2017 [38]	Surgical population, n = 138; Italy	Retrospective single center cohort study	WHO criteria	81.9%	Anemia as risk factor for in-hospital death
Pries-Heje *et al*., 2022 [6]	Non-surgical population, n = 248; Denmark	Post-hoc sub study of a randomized trial	WHO criteria	85.1%	Moderate to severe anemia was associated with higher mortality

WHO, World Health Organization; IE, infective endocarditis; ICD, International Classification of Diseases.

Most of the studies reporting anemia derived their data retrospectively from database registries, using the International Classification of Diseases (ICD) codes. The variability in prevalence between these studies is large, ranging from 10–43.5% [32, 33, 34, 35, 36]. Most likely, this represents the prevalence of chronic anemia that was already present before the episode of IE, although this is not explicitly stated in the respective publications.

Furthermore, several studies reporting prevalences of anemia did not state at all which definition of anemia was used [9, 42, 43]. However, when applying the WHO criteria of <120 g/L for women and <130 g/L for men, most studies available showed higher incidences with less variability (64–88%) [37, 38, 44].

The only study so far that focused primarily on anemia in IE [6] used data from the POET trial, a prospective randomized controlled trial on antibiotic treatment in IE [45]. Here, in 248 medically managed patients with left-sided IE after stabilization of infection, i.e. a median of 14 days after diagnosis and antibiotic treatment, the percentage of patients with anemia, according to WHO criteria was 85%. Furthermore, moderate to severe anemia was found in 29% of patients.

### 2.3 Diagnosis of Anemia

The diagnosis of anemia in patients with IE should address two key aspects: confirming its presence and determining the underlying etiology.

Hemoglobin concentration, measured in whole blood by photospectrography after lysis of the erythrocytes, is the cornerstone for the diagnosis and classification of anemia. The WHO defines anemia as a hemoglobin (Hb) level below 130 g/L in males and 120 g/L in females [41]. A further subdivision based on Hb level is used to define the severity of the anemia into three classes: mild, moderate and severe (Table 2, Ref. [41]). Still, many authors have used different cutoff values to define anemia in IE patients, which can make it difficult to compare incidences and outcomes in these studies (Table 1). Some clinicians use hematocrit (HCT) as a surrogate for Hb, however this approach is not always reliable. Hb is measured directly, while HCT is a calculated value and may be less accurate if there are errors in measuring the RBC indices [46]. Furthermore, the HCT level is dependent on plasma volume and can be affected by dehydration or intravascular volume depletion.

**Table 2.** **Hemoglobin cutoffs to define anemia severity in adults, according to WHO [41]**.

	Hemoglobin concentration (in g/L)			
	No anemia	Mild anemia	Moderate anemia	Severe anemia
Male	>130	110–129	80–109	<80
Female (non-pregnant)	>120	110–119	80–109	<80

A number of other blood tests can be performed to identify the etiology of anemia, and thus differentiate between anemia of inflammation and other forms such as iron-deficiency anemia and hemolytic anemia (Table 3, Ref. [10, 11, 47, 48, 49, 50, 51]).

**Table 3.** **Laboratory tests and the relation to the etiology of anemia [10, 11, 47, 48, 49, 50, 51]**.

	Etiology of anemia				
Laboratory test	Inflammation	Iron deficiency	Inflammation + iron deficiency	Hemolysis	Chronic kidney injury
Hemoglobin	↓	↓	↓	↓	↓
Mean corpuscular volume (MCV)	Normal	↓	↓	Normal	Normal
Mean corpuscular hemoglobine (MCH)	Normal	↓	↓	Normal	Normal
Red cell distribution width	Normal or ↑	↑	Normal or ↑	↑	Normal or ↑
Reticulocyte count	Normal	↓	Normal or ↓	↑	Normal
Ferritine	Normal or ↑	↓	Normal or ↑	Normal or ↑	Normal or ↑
Serum iron level	↓	↓	↓	Normal or ↑	↓
Transferrin	↓ or normal	↑	↓ or normal	Normal	↓ or normal
Transferrin saturation	Normal or ↓	↓	Normal or ↓	Normal or ↑	↓
Erythropoietin levels	Normal	↑	↑ or normal	↑	↓
C-reactive protein	↑	Normal	↑ or normal	Normal or ↑	Normal

↑: levels are increased. ↓: levels are decreased.

Laboratory tests that describe the shape and hemoglobin concentration of erythrocytes are often grouped as RBC indices, which usually include mean corpuscular volume (MCV), mean corpuscular hemoglobin (MCH) and red cell distribution width (RDW). The MCV and MCH in IE-associated anemia has been described as normocytic, normochromic anemia due to chronic inflammation [52, 53], although large studies describing the red cell volumes in IE are lacking. Measurement of MCV and MCH can be useful for the distinction of anemia due to inflammation versus iron deficiency, as anemia in the latter typically present as microcytic hypochromic anemia (Table 3).

RDW is usually part of the RBC indices. Besides its use for differentiation of the etiology of anemia, it has been suggested as a prognostic indicator as several IE studies observed an association between increased RDW and higher postoperative and long-term mortality [54, 55, 56].

Reticulocytes are newly formed erythrocytes and are seen as a marker of bone marrow activity and suppression. The reticulocyte rate can provide an indication of the production process of RBC by the bone marrow and is normally elevated in isolated hemolysis or acute blood loss. Anemia in IE is usually not associated with reticulocytosis, but an increased reticulocyte rate has been described in IE in several cases complicated with hemolytic anemia [13, 57, 58].

The diagnosis of anemia and the determination of the etiology can be a challenging due to multiple factors. As with HCT, Hb is a measure of whole blood and the levels can be affected by dehydration or intravascular volume depletion [59]. In such cases, anemia may be masked by normal or relatively high Hb levels, which can decline following adequate intravascular volume resuscitation.

Diagnostic algorithms based on laboratory findings have been developed to identify the underlying etiology of anemia [47]. However, the practical applicability of these algorithms in the context of IE remains uncertain, as many laboratory findings are altered in mixed etiology (Table 3). For instance, reduced serum ferritin is a sensitive indicator for iron deficiency, but its concentration increases in cases of concurrent inflammation, potentially masking the iron deficiency. An IE-specific algorithm to determine the etiology of anemia is difficult to construct due to the lack of large-scale studies on the specific biological markers of anemia in IE. In addition, prior treatment with iron supplements, Epo or RBC transfusions can interfere with biological markers for anemia, making them less useful for differentiation of the etiology. Recent RBC transfusion for instance is likely to increase the RDW.

### 2.4 Transfusion Practice in Infective Endocarditis

Transfusion practice must balance the need to support oxygen delivery with the risks of transfusion, especially in cardiovascular patients. According to the 2023 Association for the Advancement of Blood and Biotherapies (AABB) guidelines, restrictive transfusion thresholds are generally recommended, advising transfusions at a hemoglobin level below 70 g/L for most adult patients (strong recommendation, moderate certainty evidence) and below 80 g/L for those with symptomatic cardiovascular disease.

For patients undergoing cardiac surgery, clinicians may choose a threshold of 75 g/L [60]. Transfusion decisions may also consider symptom severity, clinical context, and comorbidities, especially given IE’s systemic inflammation and potential for coagulopathy.

While restrictive RBC transfusion strategies appear safe in most clinical settings [61], the results of the recently published MINT trial showed that a liberal strategy of blood transfusion might improve outcomes in patients with acute myocardial infarction (MI) and anemia [62]. Hence, further research, especially in patients with specific cardiovascular diseases such as IE is warranted.

Data on the incidence of blood transfusion in patients with IE is limited (Table 4, Ref. [6, 22, 33, 35, 38, 40, 44]). In patients treated conservatively, a transfusion rate of 14–18.5% has been published [6, 35, 40].

**Table 4.** **Transfusion rate in patients with IE**.

Author, year	Population, country	Study design	RBC transfusion rate	Comment
Non-surgical patients				
Pries-Heje *et al*., 2022 [6]	Medical population, n = 248; Denmark	Post-hoc substudy of a randomized trial	16.9%	Non-surgical cohort, after stabilization of infection
Mentias *et al*., 2020 [40]	Mixed population, n = 1868; USA	Retrospective database registry	14%	Patients with IE after TAVR
Siddiqui *et al*., 2022 [35]	Mixed population, n = 9029; USA	Retrospective database registry	18.5%	Patients with tricuspid valve IE, treated medically
Surgical patients				
Gatti *et al*., 2017 [38]	Surgical population, n = 138; Italy	Retrospective single center cohort study	65.2%	Multiple blood transfusion (>2 RBCs) 47.1%
Dahn *et al*., 2016 [44]	Surgical population, n = 92; Canada	Retrospective single center cohort study	88%	Patients undergoing aortic valve replacement with aortic regurgitation, with and without IE
Alkhouli *et al*., 2019 [33]	Surgical population, n = 34,655; USA	Retrospective database registry	38%	Patients undergoing mitral valve surgery with IE
Siddiqui *et al*., 2022 [35]	Mixed population, n = 9029; USA	Retrospective database registry	42.4%	Patients undergoing tricuspid valve surgery with IE
Breel *et al*., 2023 [22]	Surgical population, n = 31; The Netherlands	Prospective observational study	56%	42% plasma transfusion, 68% platelet transfusion

RBC, red blood cell.

In patients undergoing cardiac surgery for IE, there is significant variability in RBC transfusion rate, with studies reporting ranges from 38–88% [22, 33, 35, 38, 44]. This wide range of transfusion rates is not unique to IE and reflects a broader pattern observed in general cardiac surgery [63, 64] and intensive care units [65]. The variation in transfusion practices between different medical centers can be attributed, at least in part, to differences in institutional culture and established protocols.

In studies examining risk factors for allogeneic blood transfusion in cardiac surgery, IE was found to be an independent risk factor for RBC transfusion [66, 67]. Likewise, IE was established as risk factor for intraoperative massive transfusion (more than four units of RBC) [68], with some patients requiring more than 10 units of red blood cells intraoperatively [69].

Furthermore, patients undergoing valve replacement appear to have a larger risk of transfusion compared to those who undergo valve repair [69]. Additionally, prosthetic valve IE surgeries typically require more blood transfusions than native valve IE procedures [70]. Consistent with general cardiac surgery trends, female patients receive more RBC transfusion than their male counterparts during surgical interventions [71].

While there is little data on RBC transfusion in patients with IE, there is no available evidence on the incidence of plasma or platelet transfusion, as well as the use of coagulation factors treatment in this patient group. While this knowledge gap predominantly concerns surgical patients, it would be interesting to know whether these rates are also increased in non-surgical IE patients. Interestingly, patients who require platelet transfusion during cardiac surgery are more likely to have endocarditis [72], which suggests patients with IE are more likely to receive platelet transfusion. While there is hence a clear knowledge gap, a recently developed prediction model has been published to predict bleeding complications in patients with IE undergoing cardiac surgery. It includes four variables: platelet count, systolic blood pressure, heart failure and vegetations on mitral and aortic valve [73]. Whether such prediction models may serve to identify patients at risk and possibly limit transfusion volume requires further study.

### 2.5 Association of Anemia and Transfusion on Outcomes in Infective Endocarditis

Although literature is limited, anemia has been associated with increased mortality in IE in a number of studies (Table 5, Ref. [6, 29, 37, 38, 39, 74, 75, 76]) [6, 37, 38, 74]. Moreover, the study by Pries-Heje *et al*. [6] primarily focused on anemia in IE and demonstrated that that mortality risk increased as anemia severity worsened. Gatti *et al*. [38] identified anemia as a risk factor for mortality and incorporated it into their proposed risk scoring system (ANCLA) for predicting mortality after surgery for IE. However, a subsequent validation study of various scoring systems, including ANCLA, found that anemia was not significantly associated with in-hospital mortality. Despite this, the ANCLA scoring system still proved to be the most accurate among those tested for predicting mortality in IE patients [75].

**Table 5.** **Adverse effects of anemia in IE**.

Author, year	Population, country	Study design	Adverse effects of anemia
Pries-Heje *et al*., 2022 [6]	Non-surgical population, n = 248; Denmark	Post-hoc substudy of a randomized trial	Anemia as risk factor for 6 months and 3-year mortality. Risk increased with increased severity of anemia
Gatti *et al*., 2017 [38]	Surgical population, n = 138; Italy	Retrospective single center cohort study	Anemia as risk factor for in-hospital mortality
Gatti *et al*., 2017 [75]	Surgical population, n = 361; Italy	Retrospective multicenter cohort study	Anemia was not associated with in-hospital mortality
Lu *et al*., 2013 [37]	Mixed population, n = 148; Australia	Retrospective single center cohort study	Anemia was associated with all-cause long-term mortality
Farag *et al*., 2017 [74]	Surgical population, n = 360; Germany	Retrospective single center cohort study	Anemia as risk factor for long-term mortality
Legrand *et al*., 2013 [76]	Surgical population, n = 202; France	Retrospective single center cohort study	Preoperative anemia was associated with postoperative AKI
Yoshioka *et al*., 2015 [29]	Surgical population, n = 267; Japan	Retrospective multicenter cohort study	Anemia was associated with new intraoperative hemorrhagic stroke
Agrawal *et al*., 2020 [39]	Mixed population, n = 187,438; USA	Retrospective database registry	Anemia as risk factor for 30-day hospital readmission

AKI, acute kidney injury.

Other adverse outcomes associated with anemia in IE are postoperative acute kidney injury (AKI), intraoperative hemorrhagic stroke and hospital readmission [29, 39, 76]. The negative association between anemia and outcomes for these patients is consistent with existing literature on general cardiac surgery, where large prospective and multicenter studies have demonstrated a clear association between preoperative anemia and adverse outcomes including AKI, prolonged ventilation, increased RBC transfusion and mortality [77, 78, 79].

Although often necessary, RBC transfusion can have negative consequences for patients with IE (Table 6, Ref. [35, 76, 80]). A study by Siddiqui *et al*. [35] found an association between RBC transfusion and adverse outcomes. Other studies have demonstrated an association between intraoperative RBC transfusion with prolonged intensive care unit (ICU) stay and AKI [76, 80]. The risk of kidney function deterioration following transfusion may be significant for patients with IE, who often already have compromised renal function. However, it remains unclear whether the transfusion itself is the primary cause of the adverse effects or if the underlying conditions necessitating transfusion, such as anemia and bleeding, play a more significant role. Evidence suggests that both factors contribute. Previous research in cardiac surgery has found an association between transfusion during cardiac surgery and the risk of AKI [81] with this risk being more pronounced in anemic patients compared to non-anemic individuals [82]. A potential pathophysiological mechanism for this negative effect during surgery involves an already hypoxic kidney due to reduced oxygen delivery in anemia, combined with inflammatory mediators, oxidative stress and an excessive iron and free hemoglobin load due to transfusion [81].

**Table 6.** **Adverse effects of transfusion in IE**.

Author, year	Population, country	Study design	Adverse effects of RBC transfusion
Siddiqui *et al*., 2022 [35]	Mixed population, n = 9029; USA	Retrospective database registry	RBC transfusion as risk factor for adverse outcomes
Legrand *et al*., 2013 [76]	Surgical population, n = 202; France	Retrospective single center cohort study	RBC transfusion on day of surgery was associated with postoperative AKI
Huang *et al*., 2023 [80]	Surgical population, n = 896; China	Retrospective single center cohort study	RBC transfusion as risk factor for prolonged ICU-stay

ICU, intensive care unit.

### 2.6 Management Strategies for Anemia in Infective Endocarditis

The primary approach in managing anemia in chronic infection or inflammation is to treat the underlying disease [11]. Although outside the scope of this review, it is crucial in the treatment of IE to promptly start empiric antibiotic therapy, while awaiting identification of the pathogen [83].

There is currently no published data on the effects of pharmacological interventions for managing anemia in IE. Nevertheless, the implementation of a patient blood management (PBM) program for patients with IE may be a rational approach. Typical strategies within PBM include the administration of oral iron supplements, intravenous iron supplementation (IIS), erythropoiesis-stimulating agents (ESA), or a combination of these therapies.

Oral iron supplementation has traditionally been the standard treatment for iron deficiency. However, for patients who require rapid and effective preoperative iron replenishment, this approach may fall short. The limitations of oral iron supplementation in this context include the extended duration of therapy required, the lower bioavailability and the poor tolerance for oral iron experienced by patients [47, 48]. IIS has a higher bioavailability and is usually better tolerated. A meta-analysis of the effects of IIS in a wide range of medical and surgical specialties showed a significant increase in Hb levels and a reduction in RBC transfusion in the group treated with IIS [84]. Furthermore, a recent meta-analysis focusing on IIS in patients undergoing cardiac surgery demonstrated that IIS is effective in reducing RBC transfusion rates and increasing Hb levels postoperatively, specifically between days four to ten and after day 21. However, IIS does not appear to impact mortality, renal function, or ICU-stay [85]. Still, due to the lack of studies the efficacy of IIS in IE patients remains unclear.

On the other hand, IIS have been linked to an increased risk of infection as the elevated free serum iron may promote bacterial growth [84]. A recent meta-analysis on IIS in a mixed population found an increased risk of infection in the group treated with IIS, although the risk of bias was high in most included studies [86]. The use of IIS has been discouraged in cases of active infection in other large studies [87], although a small retrospective study found no adverse outcomes in patients with active infection treated with IIS [88]. Future trials are needed to determine the efficacy and safety of this therapy for patients with IE.

ESA have not yet been studied in patients with IE, although they have been extensively investigated in various other clinical contexts. A recent meta-analysis showed that ESA, often combined with iron suppletion, significantly reduced transfusion requirements in patients undergoing cardiac surgery [89]. However, most studies involved preoperative treatment over several days, which may not be feasible for IE patients with moderate to severe anemia requiring urgent intervention. Interestingly, some studies have shown promising results with shorter ESA treatment durations. A single administration of Epo two days before surgery has been found to effectively reduce perioperative RBC transfusion and increase Hb levels by day four [90]. Even a one-day preoperative Epo treatment combined with IIS, folic acid and vitamin B12 seems to have a positive effect on transfusion requirements, albeit with a modest mean reduction of one RBC unit [91]. This positive effect was seen in all etiologies of anemia in the study [92].

These findings may be particularly relevant in IE patients, since the etiology of anemia in IE is not fully understood and likely multifactorial. Currently, there is one study registered in the clinical trial registry investigating the treatment of ESA combined with IIS in IE [93]. Future clinical trials are warranted to establish the potential benefits of these treatments in patients with IE.

Future treatment options for IE-related anemia may include adjunctive therapies targeting the underlying mechanism of anemia. IL-6 inhibitors, like tocilizumab, have shown to inhibit hepcidin proliferation and improve anemia in autoimmune diseases with inflammation such as rheumatoid arthritis and Castleman’s disease [94, 95, 96]. However, the effects of these targeting therapies for patients with IE, where inflammation is a result of infection, remain unknown, and future research should clarify the potential benefits of these novel drugs.

**Fig. 1.**
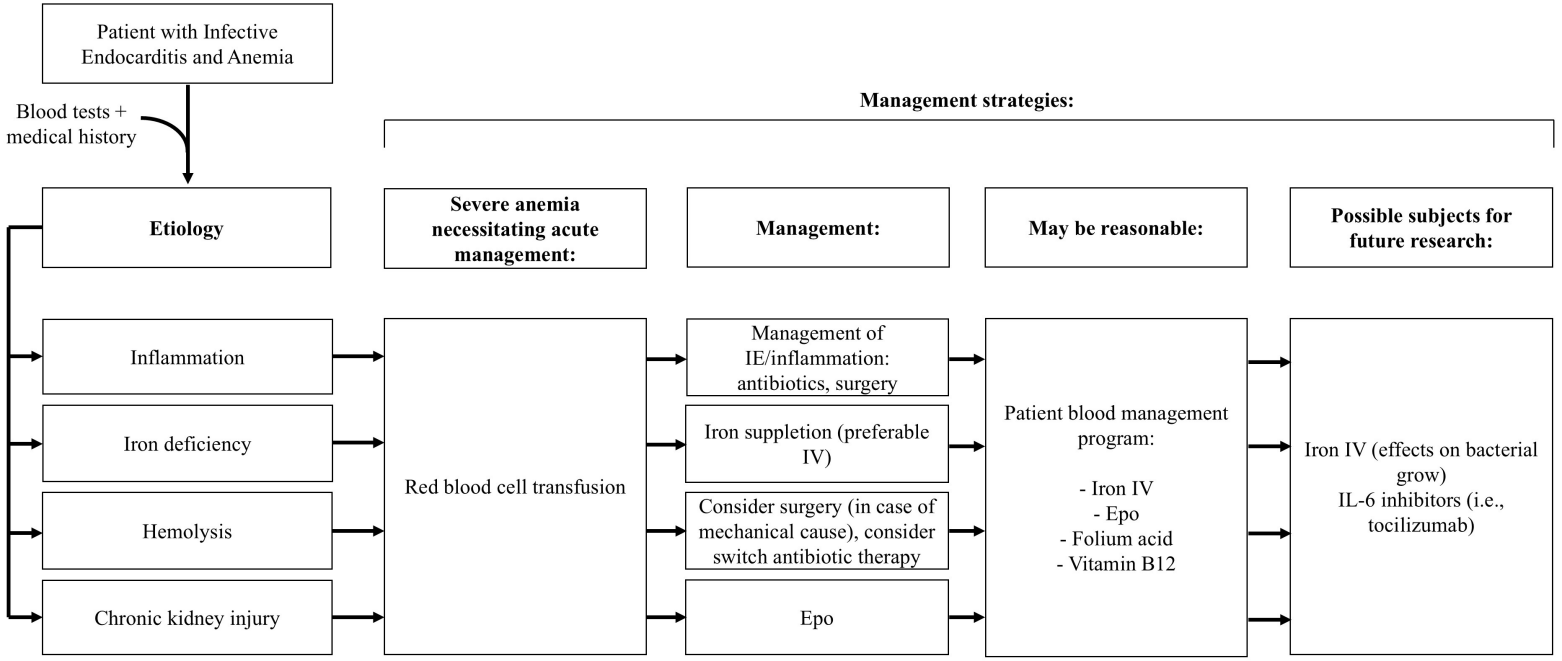
**Schematic algorithm for management strategies of anemia in infective endocarditis**. Epo, erythropoietin; IV, intravenous; IL, interleukin.

A potential algorithm for the treatment of anemia in IE is shown in Fig. 1.

## 3. Conclusion

In summary, anemia and transfusion play critical roles in the management and outcomes of IE. Anemia is highly prevalent among IE patients and is linked to increased mortality and complications, particularly in patients requiring cardiac surgery. Transfusions, often necessary due to anemia, correlate with adverse outcomes such as AKI and extended ICU stays. However, it remains unclear whether these outcomes result directly from transfusion itself or the underlying conditions that necessitate it.

Management strategies for anemia in IE, including IIS and ESA, have shown promising preliminary results in reducing transfusion needs in patients without IE, yet their efficacy and safety in patients with active IE has not been proved. Future research is essential to determine safe, effective management protocols for this vulnerable patient group.
